# Activity-dependent adaptations in inhibitory axons

**DOI:** 10.3389/fncel.2013.00219

**Published:** 2013-11-21

**Authors:** Cátia P. Frias, Corette J. Wierenga

**Affiliations:** Division of Cell Biology, Faculty of Science, Utrecht UniversityUtrecht, Netherlands

**Keywords:** GABAergic synapses, interneurons, homeostatic plasticity, axons, cell adhesion molecules

## Abstract

Synaptic connections in our brains change continuously and throughout our lifetime. Despite ongoing synaptic changes, a healthy balance between excitation and inhibition is maintained by various forms of homeostatic and activity-dependent adaptations, ensuring stable functioning of neuronal networks. In this review we summarize experimental evidence for activity-dependent changes occurring in inhibitory axons, in cultures as well as *in vivo*. Axons form many presynaptic terminals, which are dynamic structures sharing presynaptic material along the axonal shaft. We discuss how internal (e.g., vesicle sharing) and external factors (e.g., binding of cell adhesion molecules or secreted factors) may affect the formation and plasticity of inhibitory synapses.

## INTRODUCTION

Our brain is a complex organ with tremendous self-organizing abilities. Its computational power is based in the adjustable synaptic connections between neurons. When new experiences and memories are established, specific synapses in specific brain regions are changed, both in strength and in number. To ensure proper global functioning despite changes in local connectivity, these synaptic changes must be coordinated within neurons, as well as within neuronal circuits. An important aspect is the coordination between changes in excitatory and inhibitory synapses to regulate and maintain an overall balance between excitation and inhibition. When this balance is disturbed, neurological diseases such as autism or schizophrenia can develop (Palop et al., 2007; Yizhar et al., 2011; Han et al., 2012; Bateup et al., 2013).

Homeostatic plasticity is a term that is used for plasticity mechanisms which ensure that overall neuronal spiking activity is maintained within neuronal networks. Many forms of homeostatic plasticity have been described in excitatory and inhibitory neurons (Turrigiano, 2008; Wenner, 2011; Pozo and Goda, 2010; Tyagarajan and Fritschy, 2010). In neuronal circuits in the brain, inhibitory neurons serve multiple functions, making connections to excitatory as well as inhibitory neurons, and providing feedforward inhibition to some neurons, while supplying feedback input to others. In such complicated networks, there are multiple ways to compensate for changes in network activity, which makes it hard, if not impossible, to classify synaptic changes in inhibitory axons as truly homeostatic. Therefore, we will discuss activity-dependent feedback signals in inhibitory axons in a more general context in this review. We will discuss experimental evidence showing that synaptic activity can affect the formation and plasticity of inhibitory synapses and we will speculate on possible mechanisms.

## ACTIVITY-DEPENDENT ADAPTATIONS OF INHIBITORY SYNAPSES

When prolonged changes occur in network activity, homeostatic mechanisms come into play which adjust excitatory and inhibitory synapses to compensate and restore the activity level in the network (Turrigiano, 1999, 2011; Burrone and Murthy, 2003; Pozo and Goda, 2010; Wenner, 2011). Generally speaking, when the activity is too high, excitation must be downregulated, and inhibition should be increased to bring activity levels back to baseline. And opposite changes should occur during activity blockade. Homeostatic plasticity has been studied extensively in cultures, where neurons are randomly connected. Dissociated cultures provide superb access for experimental manipulations and therefore form an excellent system to study the cell biological mechanisms underlying homeostatic plasticity. However, in our brain neurons are embedded in multiple neuronal networks and make specific synaptic connections. Recurrent connections between neurons or groups of neurons are very common and different types of GABAergic interneurons are known to have high specificity, making inhibitory synapses onto specific target neurons, including inhibitory neurons (Pfeffer et al., 2013; Jiang et al., 2013). In such complex networks, it is not easy to determine rules of homeostatic plasticity. Adaptation to changes in the activity of the network will be strongly synapse-specific and likely depends on the precise function and location of the synapse in the network (Chen et al., 2011; Maffei et al., 2004; Maffei and Turrigiano, 2008). Here we briefly describe the experimental evidence for activity-dependent plasticity of inhibitory synapses from *in vitro* (i.e., in dissociated and organotypic cultures) and *in vivo* studies.

### Primary cell cultures

Activity manipulations in cultures of dissociated hippocampal or neocortical neurons generally affect excitatory and inhibitory synapses in opposite directions. After a prolonged period of activity blockade, excitatory synapses get strengthened and inhibitory synapses are weakened, and synaptic changes are in opposite directions when activity is enhanced (Turrigiano et al., 1998; Kilman et al., 2002; Hartman et al., 2006; Swanwick et al., 2006). Therefore, changes in excitation and inhibition cooperate to compensate for the change in activity level. For inhibitory synapses, changes in mIPSC amplitude are most commonly reported, reflecting changes in synaptic strength. Sometimes they are accompanied by changes in mIPSC frequency, which could either reflect a change in the number of synapses or a change in release properties. Dissociated cultures provide excellent experimental access and are therefore well-suited for studying underlying mechanisms of homeostatic plasticity. However, the artificial environment in which neurons grow in culture may affect synaptic maturation (Wierenga et al., 2006; Rose et al., 2013) and consequently cellular or synaptic mechanisms of plasticity. Cellular mechanisms that were identified to mediate the changes in inhibitory synapses after activity manipulations include: changes in number of postsynaptic receptors (Kilman et al., 2002; Swanwick et al., 2006; Saliba et al., 2007; Peng et al., 2010; Rannals and Kapur, 2011) or scaffolding proteins (Vlachos et al., 2012; study in slice cultures) on the postsynaptic side, and changes in presynaptic release probability (Kim and Alger, 2010), presynaptic vesicle loading (De Gois et al., 2005; Hartman et al., 2006; Lau and Murthy, 2012), or GABA-producing enzymes (Peng et al., 2010; Rannals and Kapur, 2011) on the presynaptic side. Only in a few cases, changes in the number of inhibitory synapses were reported (Hartman et al., 2006; Peng et al., 2010). Homeostatic changes of inhibitory synapses could be induced in a cell autonomous fashion (Peng et al., 2010), or required a change in activity of the entire neuronal network (Hartman et al., 2006), emphasizing that there are multiple mechanisms of homeostatic plasticity at inhibitory synapses. In particular, distinct mechanisms could exist for activity-dependent downregulation and upregulation of inhibitory synapses.

### Organotypic cultures

In contrast to dissociated cultures neurons in more intact tissue, such as acute slices or organotypic cultures, make more specific connections and form structured networks. This network configuration makes the interpretation of synaptic changes more complex. In slices that were submitted to activity manipulations, changes in inhibition have been observed opposite to (Marty et al., 2004; Karmarkar and Buonomano, 2006; Kim and Alger, 2010) as well as in conjunction with (Buckby et al., 2006; Echegoyen et al., 2007) changes in excitation. It was also shown that different types of homeostatic mechanisms have different time courses (Karmarkar and Buonomano, 2006) and that different subsets of inhibitory synapses can respond differently. For instance, the presence of cannabinoid receptors in a subset of inhibitory synapses renders them selectively receptive to changes in endocannabinoid levels induced by inactivity (Kim and Alger, 2010). In another example, inactivity differentially affected somatic and dendritic inhibitory inputs on CA1 pyramidal cells. Interestingly, both types of synapses showed reduction in the number of presynaptic boutons and upregulation of release probability, but the functional end-effect on inhibitory input to the postsynaptic cells was different (Chattopadhyaya et al., 2004; Bartley et al., 2008). This emphasizes that simple *in vitro* homeostatic rules for scaling inhibitory synapses get complicated in more complex networks. In addition, other factors such as different cell (glia) types or the extracellular environment in more intact tissue potentially influence homeostatic plasticity compared to dissociated cultured cells.

### In vivo studies

Typically, when studying activity-dependent or homeostatic changes *in vivo*, sensory deprivation is used as experimental paradigm to lower activity levels in the primary sensory cortex (e.g., whisker trimming, monocular deprivation, or retinal lesion). While *in vitro* activity manipulations by pharmacological means affect the activity of all neurons in equal amounts, sensory deprivation *in vivo* will affect different neurons in the circuitry differentially. Therefore, *in vivo* responses of inhibitory synapses to changes in activity vary widely and strongly depend on the specific cell types, cortical layer, and specific circuitry (Maffei et al., 2004; Maffei and Turrigiano, 2008; Chen et al., 2011). Furthermore, it is well-known that inhibition in sensory cortex areas undergoes important developmental changes (Hensch, 2005), which means that the same deprivation paradigm can have different effects on inhibitory synapses depending on the postnatal period that is considered (Chattopadhyaya et al., 2004; Maffei et al., 2006; Maffei et al., 2010). An emerging theme from the* in vivo* studies is that inhibitory synapses can respond rapidly to sensory deprivation. It was shown that inhibitory axons in cortical layer 2/3 reduce the number of boutons within the first 24 h after a retinal lesion or monocular deprivation (Chen et al., 2011; Keck et al., 2011). Over longer periods, inhibitory axons in the barrel cortex were shown to sprout and form new axonal branches after whisker plucking (Marik et al., 2010). Interestingly, the reduction of inhibition was often found to precede adaptive changes of the excitatory circuitry (Marik et al., 2010; Keck et al., 2011). The rapid downregulation of inhibition might serve to render the local circuit more permissive for excitatory plasticity to occur (Ormond and Woodin, 2011; Gambino and Holtmaat, 2012). In two recent studies it was shown that inhibitory synapses that are located on spines (presumably next to an excitatory synapse) showed much higher turnover rates compared to inhibitory synapses on shaft after visual deprivation (Chen et al., 2012; vanVersendaal et al., 2012). It will be interesting to see whether direct cross talk of the two types of synapses exists.

In conclusion, there is a large amount of compelling evidence for activity-dependent adaptations in inhibitory synapses *in vitro* as well as *in vivo*. The precise expression mechanisms significantly vary between different preparations and experimental paradigms.

## AXONS

In this review we focus on possible feedback signals that occur in inhibitory axons in response to changes in network or synaptic activity and that induce changes in the number or properties of presynaptic terminals along the axon. The axon of a single neuron forms several thousands of presynaptic terminals (i.e., ``boutons'') along its shaft and contacts many different postsynaptic neurons. Presynaptic boutons along an axon show a large variety in their volumes, in the number of synaptic vesicles and in the presence or absence of mitochondria (Shepherd and Harris, 1998). It is now well-established that neighboring boutons are not independent entities, but they continuously share and exchange molecular components of the release machinery and synaptic vesicles (Krueger et al., 2003; Darcy et al., 2006; Sabo et al., 2006; Staras, 2007; Yamada et al., 2013). Synaptic vesicles may not belong to a specific presynaptic terminal, but form a super pool of vesicles in the axonal shaft and are shared by multiple release sites (Staras et al., 2010).

The exchange of presynaptic proteins means that the exact composition of presynaptic terminals is continuously changing. These changes can occur in a correlated fashion with the postsynaptic site in some synapses, but can be uncoordinated in others (Fisher-Lavie et al., 2011; Fisher-Lavie and Ziv, 2013). Release properties and synaptic strength are highly variable between individual boutons along the same axon (Branco et al., 2008; Zhao et al., 2011; Rose et al., 2013). Therefore the demand for synaptic vesicles or other presynaptic proteins will vary between presynaptic boutons and neighboring boutons compete for available resources. Indeed, reduced availability of synaptic proteins within the axon has been shown to enhance competition between boutons (Yamada et al., 2013). In addition, vesicle exchange is regulated by neuronal activity through changes in axonal calcium levels (Kim and Ryan, 2013, 2010).

Synaptic vesicles are kept at the presynaptic terminal by interacting with a scaffolding meshwork of actin, β-catenin, synapsin, and many other proteins (Bamji et al., 2003; Takamori et al., 2006; Cingolani and Goda, 2008; Fernández-Busnadiego et al., 2010; Peng et al., 2012; Taylor et al., 2013). Synaptic vesicles can escape from the presynaptic terminal into the axon, while other vesicles that were traveling along the axonal shaft can be captured. Although the loss of a strict presynaptic compartmentalization may seem disadvantageous at first, the main advantage of sharing presynaptic material between boutons is flexibility. When presynaptic material is continuously being lost and gained at synapses, synapses can rapidly change their strength by adjusting the ratio of vesicle capture and release (Wu et al., 2013). In addition, synapses can be formed or disassembled within a few hours. It was shown that presynaptic proteins can be transported together in small packages in axons (Friedman et al., 2000; Zhai et al., 2001; Wu et al., 2013). Such multi-protein packages can be recruited to locations where new synapses are being formed and a few of these ready-to-go packages are enough to rapidly build a functional active zone and release site (Jin and Garner, 2008; Owald and Sigrist, 2009).

Live imaging of axons have shown that transient and mobile release sites exist (Krueger et al., 2003) and that transient boutons occur at predefined locations along the axon (Sabo et al., 2006; Ou and Shen, 2010; Bury and Sabo, 2011), presumably reflecting contact sites with potential postsynaptic targets (Wierenga et al., 2008; Schuemann et al., 2013). The transient nature of boutons in such locations suggest that presynaptic structures are immature or incomplete and may serve a role in ``testing'' a new synaptic location (Wierenga et al., 2008; Dobie and Craig, 2011; Fu et al., 2012; Schuemann et al., 2013). Transient boutons might therefore reflect failed attempts or intermediate stages of building new synapses, but they could also have a physiological function. Transient boutons, or orphan release sites, are likely capable of neurotransmitter release (Krueger et al., 2003; Coggan et al., 2005; Ratnayaka et al., 2011) and besides having a role in synapse formation, ectopic release of neurotransmitter by transient boutons could also serve to signal to nearby astrocytes or to regulate ambient neurotransmitter levels.

Synapse assembly is a complicated process involving interactions of multiple proteins. It does not necessarily need to be a linear process, where one component necessarily recruits the next, but some of the interactions could occur in parallel and the sequence of protein recruitment may vary. Rapid self-assembly of presynaptic components may be an important element during synaptogenesis. This would mean that the formation of a presynaptic terminal merely needs an initial trigger to ascertain a specific axonal location or postsynaptic partner, but then the new presynaptic terminal ``unfolds'' automatically by spontaneous clustering of its components. It is likely that multiple triggers can induce self-assembly. Indeed, it was recently reported that synaptic material is actively prevented from aggregating and assembling new synapses during transport (Wu et al., 2013), supporting the self-assembly hypothesis. Without prevention of aggregation, presynaptic terminals were formed at locations where no postsynaptic targets were present and no postsynaptic specializations were recruited. Furthermore, the ectopic formation of presynaptic terminals on non-neuronal cells can be induced when these cells express ``synaptogenic'' cell adhesion molecules (Scheiffele et al., 2000; Graf et al., 2004; Takahashi et al., 2012), indicating that a single trans-synaptic trigger is enough to start the presynaptic cascade to assemble functional release sites.

**FIGURE 1:**
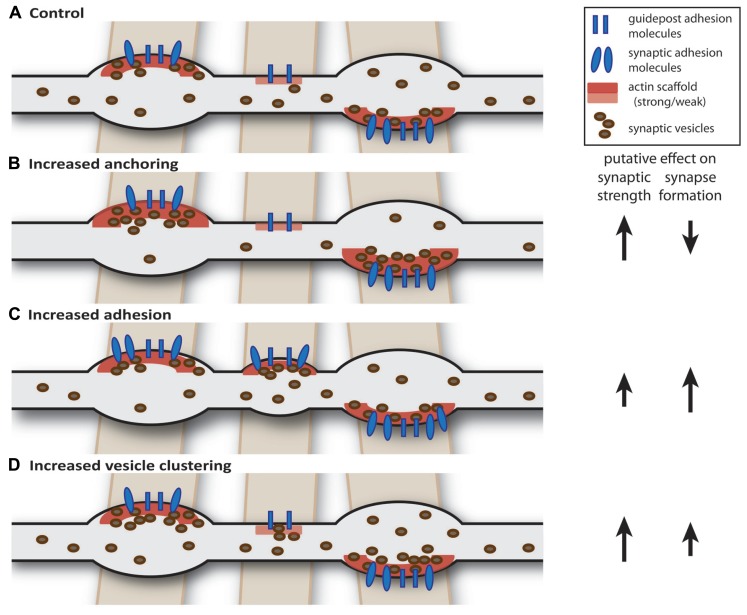
**Intrinsic factors: axon-wide increase in synaptic strength or release properties may also affect synapse formation.** **(A)** Schematic drawing of an axon (gray) forming two mature and one nascent bouton on crossing dendrites (brown). We hypothesize that axon-dendrite crossings are marked at potential synaptic locations and contain guidepost adhesion molecules (Shen and Bargmann, 2003; Shen et al., 2004) and weak actin scaffold (Chia et al., 2012). **(B)** Increasing anchoring of vesicles at presynaptic terminals could decrease the pool of ``free'' vesicles, thereby reducing the probability of forming new synapses (Yamada et al., 2013). **(C)** Increasing synaptic adhesion increases the number of synapses (Scheiffele et al., 2000; Takahashi et al., 2012; Kuzirian et al., 2013) and may also affect properties of existing synapses (Varoqueaux et al., 2006; Wittenmayer et al., 2009). **(D)** Overexpression of vesicle clustering factors induce changes in release properties, but may also promote synapse formation (Wentzel et al., 2013; Wu et al., 2013).

A dynamic control of the strength and number of presynaptic terminals in axons implies that control of transport, capture, and release of synaptic material are essential processes regulating the formation, maintenance, and strength of presynaptic terminals. In a dynamic axon with competing presynaptic terminals, a general change in synaptic strength is expected to also have an effect on ongoing synapse formation within the same axon and vice versa (**Figure 1**). For instance, enhancement of synaptic strength by increasing vesicle capture or anchoring at presynaptic terminals would also result in lower amounts of ``free'' vesicles in the axonal shaft thereby reducing the chance that new synapses are formed at nascent sites (Yamada et al., 2013). However, a similar increase in synaptic strength could also be achieved by increasing vesicle clustering (Wu et al., 2013), but such a mechanism would actually promote synapse formation (**Figure 1**). This illustrates that presynaptic plasticity and synapse formation should be considered mutually dependent processes when neighboring presynaptic terminals are sharing synaptic proteins and vesicles.

### INHIBITORY AXONS

Most of the studies that were mentioned above were performed in excitatory axons and it is not entirely clear to what extent the results are also valid for inhibitory axons. Important observations have been made in live imaging studies of inhibitory axons. Presynaptic terminals in inhibitory axons were shown to be dynamic structures* in vitro* and* in vivo*. Inhibitory boutons can appear, disappear, and reappear over the course of several minutes to hours (Kuhlman and Huang, 2008; Marik et al., 2010; Keck et al., 2011; Fu et al., 2012; Schuemann et al., 2013), and the same has been shown for clusters of pre- or postsynaptic proteins at inhibitory synapses (Dobie and Craig, 2011; Chen et al., 2012; Kuriu et al., 2012; vanVersendaal et al., 2012). Bouton dynamics are comparable *in vitro* and *in vivo* and likely reflect physiological processes. Interestingly, these dynamic changes were shown to be affected by network activity and mediated, at least in part, by activation of GABA receptors (Fu et al., 2012; Kuriu et al., 2012; Schuemann et al., 2013). This could represent a mechanism by which the synaptic activity of inhibitory synapses may regulate their own stability using GABA as a feedback signal.

New inhibitory synapses can form rapidly by the appearance of a bouton at locations where the inhibitory axon is in close contact with a dendrite, without the involvement of dendritic protrusions (Wierenga et al., 2008; Dobie and Craig, 2011). This finding indicates an important contrast with the formation of excitatory synapses, in which new synapses are usually formed by the outgrowth of dendritic protrusions. It also emphasizes the important role of crosstalk between neighboring boutons within inhibitory axons for synapse formation. Nascent inhibitory synapses recruit release machinery proteins and synaptic vesicles on the presynaptic side and receptors and scaffolding molecules on the postsynaptic side within a few hours (Wierenga et al., 2008; Dobie and Craig, 2011; Kuriu et al., 2012; Schuemann et al., 2013). Interestingly, simultaneous translocations of pre- and postsynaptic proteins over several micrometers were observed in cultures (Dobie and Craig, 2011; Kuriu et al., 2012) and it will be interesting to see if such movement of inhibitory synapses can also occur in slices or *in vivo*. Together, these observations reveal the dynamic nature of inhibitory axons and strongly suggest that the exchange of presynaptic material between existing and emerging boutons within the axonal shaft plays an essential role in the activity-dependent formation, maintenance and plasticity of inhibitory synapses.

In general, it is not clear if molecular differences exist between excitatory and inhibitory axons, other than the neurotransmitter that is produced and loaded into synaptic vesicles. For instance, the extent or regulation of dynamic exchange between boutons could be different in these two types of axons. The protein composition of the release machinery at excitatory and inhibitory presynaptic terminals is surprisingly similar, although small difference have been reported (Gitler et al., 2004; Kerr et al., 2008; Kaeser et al., 2009; Grø, 2010; Zander et al., 2010; Boyken et al., 2013; Bragina et al., 2013). It is currently not known if some of these differences have consequences for plasticity or presynaptic dynamics within axons. Furthermore, it is not known if there are differences between axons of the various inhibitory cell types (Ascoli et al., 2008; Klausberger and Somogyi, 2008). However, there is a clear difference between excitatory and inhibitory axons in the expression of specific cell adhesion molecules at excitatory and inhibitory synapses.

### ROLE OF CELL-ADHESION MOLECULES IN SYNAPTIC PLASTICITY

The observation that inhibitory boutons appear at specific, predefined locations along the axon (Sabo et al., 2006; Wierenga et al., 2008; Schuemann et al., 2013), strongly suggests that something is marking these locations prior to bouton formation (Shen and Bargmann, 2003; Shen et al., 2004). Inhibitory axons are characterized by their tortuous and highly branched morphology and they are in close contacts with many nearby dendrites. In fact, it was shown that inhibitory axons have substantially larger overlap with the dendritic trees of their potential target neurons than expected from chance, whereas this is not the case for excitatory axons (Stepanyants et al., 2004). This suggests that inhibitory axons possibly search for or are attracted by dendrites during development. Contacts between dendrites and inhibitory axons could be maintained by guidepost cell-adhesion molecules, even without inhibitory synapses present (Shen and Bargmann, 2003; Shen et al., 2004). Their presence would mark the location of a postsynaptic dendrite and therefore a potential spot for an inhibitory synapse.

**FIGURE 2:**
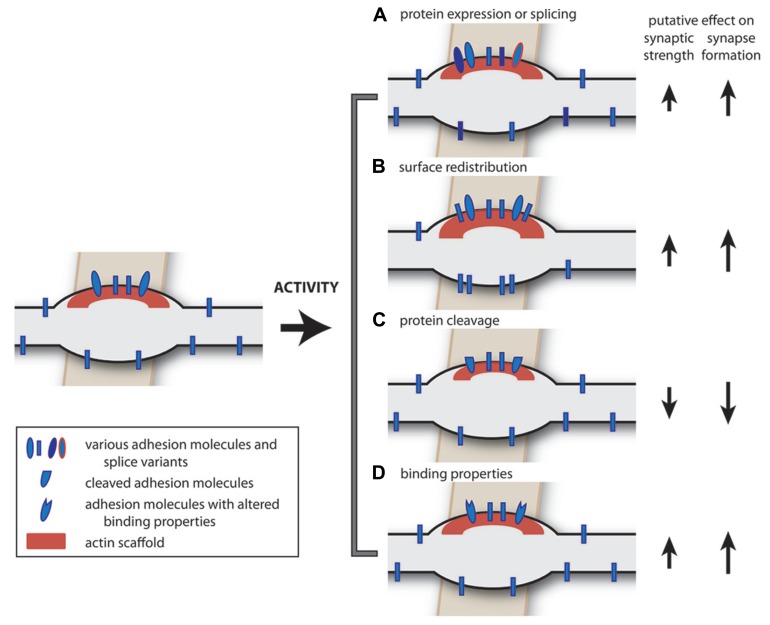
**Extrinsic factors: possible activity-dependent changes in cell adhesion molecules.** Neural activity may induce a number of changes in adhesion molecules. **(A)** The expression level of cell adhesion molecules (Cingolani et al., 2008), or their splice variants (Chih et al., 2006; Graf et al., 2006), can be regulated in an activity-dependent manner, potentially affecting synapse formation, and synapse specificity. **(B)** Activity-dependent redistribution of adhesion molecules over the axonal membrane can facilitate synapse formation (Fu and Huang, 2010). **(C)** Activity-dependent cleavage of synaptic adhesion molecules could induce synapse disassembly or plasticity (Matsumoto-Miyai et al., 2009; O'Connor et al., 2009; Peixoto et al., 2012; Suzuki et al., 2012). **(D)** Activity-dependent changes in binding properties of adhesion molecules (Kim et al., 2011a,b) could affect synaptic properties. In addition, the intracellular signaling pathways (not depicted) may also be regulated in an activity-dependent manner, affecting all of these processes.

Cell adhesion molecules are transmembrane proteins, which play a role in recognition of synaptic partners during the initial contact and provide specificity of synaptic connections (Meijers et al., 2007; Wojtowicz et al., 2007). In addition, cell adhesion molecules have been shown to play a role in the process of synaptic maturation following the initial contact, in the recruitment of synaptic proteins as well as in maintaining proper synaptic function throughout the lifetime of the synapse (Dalva et al., 2007; Krueger et al., 2012; Thalhammer and Cingolani, 2013). Cell adhesion molecules can also play an active role in the process of synapse disassembly (O'Connor et al., 2009). In conclusion, cell adhesion molecules are an essential part of synapses and synaptic plasticity most likely involves regulation of cell-adhesion molecules. Here we discuss how synaptic adhesion could be regulated in an activity-dependent manner (**Figure 2**) and we summarize current knowledge of cell adhesion molecules that are specific for inhibitory synapses.

#### Activity-dependent regulation of protein expression levels

Cell adhesion molecules often serve as recognition or identity signals to specify neuronal connectivity, and they can either promote or prevent synapse formation (Dalva et al., 2007; Bukalo and Dityatev, 2012). Neurons presumably express a combination of cell adhesion molecules and the specific combination (both the variety as well as relative levels) likely regulate the specificity and number of their synaptic contacts (Sassoè-Pognetto et al., 2011). Different cell adhesion molecules can cooperate to promote synapse formation, but the opposite is also possible: cis-interactions between different cell adhesion molecules within a neuron can preclude trans-interactions with cell adhesion molecules on neighboring neurons and thereby inhibit or prevent synapse formation (Taniguchi et al., 2007; Lee et al., 2013). Most importantly, the combination of cell adhesion molecules that a neuron expresses might not be static (**Figure 2A**). Indeed, for a number of cell adhesion molecules, activity-dependent changes in expression level have been observed (Pinkstaff et al., 1998; Cingolani et al., 2008; Pregno et al., 2013). Changes in expression level may be regulated by the activity level of the neuron itself or by extracellular signals from the environment, such as secreted factors from neighboring cells. For instance, TNFα, a glia-derived factor, which is secreted in an activity-dependent manner, regulates expression levels of β3 integrin and N-cadherin (Kubota et al., 2009; Thalhammer and Cingolani, 2013). In theory, local protein synthesis in the axon could also contribute to changes in expression level of cell adhesion proteins (Taylor et al., 2009, 2013; Zivraj et al., 2010), but direct experimental evidence is currently lacking.

#### Activity-dependent regulation of splicing

For many adhesion molecules different splice forms have been identified. Different splice variants often have different affinities for their binding partners and thereby differentially affect synapse formation or plasticity (Missler and Südhof, 1998; Hattori et al., 2008; Aoto et al., 2013). For instance, alternative splicing of neuroligins and neurexins affects specificity for excitatory or inhibitory synapses (Chih et al., 2006; Graf et al., 2006). Therefore, alternative splicing might be a way to enlarge the available set of adhesion molecules within a neuron and to enhance the range of molecular specificity of synaptic connections.

#### Activity-dependent regulation of cell surface distribution

To have their effect specifically at synapses, cell adhesion molecules should be enriched at synaptic membranes. There is experimental evidence that the distribution of cell adhesion molecules over the cellular surface can be regulated (Tai et al., 2007; Fu and Huang, 2010). For instance, while neurexin1α shows a diffuse pattern along the axonal membrane in inhibitory axons, neurexin1β is specifically enriched in the membrane at presynaptic terminals. Anchoring of neurexin1β at presynaptic boutons is regulated by presynaptic GABA release and subsequent GABA_B_ receptor activation (Fu and Huang, 2010). Further investigation is needed to understand how such local changes are regulated by protein modifications or localized endo- or exocytosis and how they affect local synapse formation (**Figure 2B**).

#### Activity-dependent regulation of protein cleavage

Synaptic adhesion molecules execute their function by binding to a trans-synaptic partner at their extracellular domain. In some cases, the extracellular domain can be cleaved, with strong effects on local synaptic adhesion. For instance, activity-dependent cleavage of agrin was shown to mediate the formation of dendritic filopodia (Frischknecht et al., 2008; Matsumoto-Miyai et al., 2009) and cleavage of neuroligin-1 was shown to regulate synaptic strength of individual excitatory synapses in an activity-dependent manner (Peixoto et al., 2012; Suzuki et al., 2012). Many other adhesion molecules have known cleavage sites and it will be interesting to see whether this mechanism for activity-dependent regulation is also present at inhibitory synapses (**Figure 2C**).

#### Activity-dependent regulation of binding

For some cell adhesion molecules activity can regulate the binding properties of the proteins. For instance, interactions between cadherins are affected by extracellular calcium concentrations (Kim et al., 2011b) and integrins can switch between an active or inactive configuration by extra- or intracellular factors (Hynes, 2002). In this way, synaptic adhesion can be modulated in an activity-dependent manner without a change in synaptic composition (**Figure 2D**).

#### Activity-dependent regulation of interacting proteins

Upon binding to other cell adhesion molecules, cell adhesion molecules cluster at the cell membrane and signal through interactions with many intracellular proteins, whose levels may be regulated in an activity-dependent manner. Ultimately, signaling through synaptic adhesion molecules in the presynaptic terminal result in direct or indirect alterations of the actin cytoskeleton and vesicle recycling, affecting the number, function, and/or stability of synapses (Zhang et al., 2001; Tabuchi et al., 2002; Swiercz et al., 2008; Sun and Bamji, 2011; Takahashi and Craig, 2013). It will be crucial to identify the precise molecular pathways that are involved to fully understand how activity-dependent changes at inhibitory synapses occur.

## CELL ADHESION MOLECULES AT INHIBITORY SYNAPSES

The list of known synaptic adhesion molecules is rapidly growing, but our knowledge on the precise function of most of these proteins remains incomplete. Interestingly, several synaptic cell-adhesion molecules have been reported to be specifically involved in inhibitory, and not excitatory, synapses. These include sema4D (Paradis et al., 2007; Kuzirian et al., 2013), slitrk3 (Takahashi et al., 2012), and neuroligin-2 (Varoqueaux et al., 2004; Patrizi et al., 2008; Poulopoulos et al., 2009), and it is to be expected that new discoveries will be made in the near future. Here we briefly summarize what is known on the role of various cell adhesion molecules at inhibitory synapses.

### NEUROLIGIN-2

Postsynaptic neuroligins and their presynaptic partners neurexins are transmembrane cell adhesion molecules that have been established as important synaptic regulators (Südhof, 2008; Siddiqui and Craig, 2011; Krueger et al., 2012). When expressed in non-neuronal cells, neurexins as well as neuroligins can induce the formation of synapses in co-cultured neurons (Graf et al., 2004; Kang et al., 2008). This suggests that neurexins and neuroligins function in the initial assembly of synaptic connections. However, knock out studies showed that they are not strictly required for synaptogenesis, but they play a crucial role in the proper assembly and functional maturation of synapses (Varoqueaux et al., 2006). Neuroligin-2 localizes specifically to the postsynaptic membrane of inhibitory synapses (Varoqueaux et al., 2004; Chubykin et al., 2007) and has been shown to be a regulator of inhibitory synapse formation and function (Varoqueaux et al., 2006; Chubykin et al., 2007; Poulopoulos et al., 2009). Interestingly, a recent report suggested that the preferential localization of neuroligin-2 at inhibitory synapses can be contributed to the low abundance of β-neurexin1 in inhibitory axons (Futai et al., 2013), suggesting that the presynaptic axon determines specificity of cell adhesion molecules at inhibitory synapses. Mice lacking neuroligin-2 show impairments in inhibitory synaptic transmission and exhibit anxiety-like behavior and increased excitability (Blundell et al., 2009; Gibson et al., 2009; Jedlicka et al., 2011). Interestingly, although neuroligin-2 is present at all inhibitory synapses, only perisomatic synapses were affected in the absence of neuroligin-2 (Gibson et al., 2009). Recently, two adhesion molecules were found to show specific interactions with neuroligin-2 at inhibitory synapses. MDGA1 inhibits the interaction between neuroligin-2 and neurexins and therefore specifically suppresses the inhibitory synaptogenic activity of neuroligin-2 (Lee et al., 2013; Pettem et al., 2013). IgSF9 specifically localizes at inhibitory synapses on inhibitory neurons, where it binds to neuroligin-2 via the scaffolding protein S-SCAM (Woo et al., 2013). These findings raises the possibility that neuroligin-2 serves different functions at different inhibitory synapses, depending on its interactions with other cell adhesion molecules.

### SLITRK3

Leucine-rich repeat (LRR) proteins have received considerable research attention recently. The members of the subfamily of Slitrk (Slit and Trk-like) proteins are involved in synapse formation and has been linked to several neurological disorders (Aruga and Mikoshiba, 2003; Takahashi and Craig, 2013). Slitrk3 has been shown to be present at the postsynaptic side of inhibitory synapses and it can induce the formation of inhibitory synapses through its interaction with the presynaptic tyrosine phosphatase receptor PTPδ (Takahashi et al., 2012; Yim et al., 2013). Here, the specificity for inhibitory synapses is dictated by the postsynaptic slitrk3, as it was shown that presynaptic PTPδ can interfere with other synaptic organizing molecules to promote formation of excitatory synapses (Yoshida et al., 2011, 2012). The slitrk3 knock out mouse has no gross defect in brain morphology, but shows decreased expression of inhibitory markers (Takahashi et al., 2012). Accordingly, these mice have an increased susceptibility for seizures and sometimes display spontaneous seizures. Interestingly, not all inhibitory synapses were equally affected by the loss of slitrk3. In the hippocampal CA1 region, specifically inhibitory synapses in the middle of the pyramidal layer were lost (Takahashi et al., 2012). It will be interesting to examine whether specificity of inhibitory synapses correlates with different subsets of pre- or postsynaptic neurons types or function.

Members of the closely related subfamily of leucine-rich transmembrane proteins (LRRTMs) have also been implicated in excitatory synapse formation and plasticity (Linhoff et al., 2009; Ko et al., 2011; de Wit et al., 2013; Siddiqui et al., 2013), but so far no LRRTM that is specific for inhibitory synapses has been identified.

### SEMAPHORIN-4D

Semaphorins are well-known as (repulsive) axon guidance molecules acting through rearrangements of the cytoskeleton in the growth cone. They play an important role in the early development of the brain (Pasterkamp, 2012). Some semaphorins are also expressed later in development and have been implicated in the formation and plasticity of neuronal connections (Sahay et al., 2005; Morita et al., 2006; Paradis et al., 2007; O'Connor et al., 2009; Ding et al., 2012; Mizumoto and Shen, 2013). Knocking down the membrane-bound semaphorin Sema4D was shown to specifically reduce the number of inhibitory synapses, while excitatory synapses were not affected (Paradis et al., 2007). Furthermore, application of soluble Sema4D was able to increase the density of GABAergic synapses within 30 min in rat hippocampal neurons (Kuzirian et al., 2013). These new inhibitory synapses became functional within 2 h and could restore normal levels of activity in an *in vitro* model for epilepsy (Kuzirian et al., 2013). The effect of sema4D on inhibitory synapses depends on the plexinB1 receptor (Kuzirian et al., 2013). It was earlier shown that activation of plexinB1 by sema4D can induce opposing responses on the cytoskeleton, depending on different interacting proteins (Basile et al., 2004; Swiercz et al., 2008; Tasaka et al., 2012), but the intracellular pathway used for inhibitory synapse formation is not known. Sema4D is a membrane-bound protein, but the protein can also be cleaved (Basile et al., 2007; Zhu et al., 2007). It was recently shown that extracellular cleavage of sema4D occurs in neurons, but does not interfere with its synaptogenic properties at inhibitory synapses (Raissi et al., 2013).

### OTHER CELL ADHESION MOLECULES

There are many other cell adhesion molecule proteins and with continued research on inhibitory synapses, it is expected that more of them will be found to play a role at inhibitory synapses. Here we just mention a few that have been reported at inhibitory synapses.

Neural cell adhesion molecule (NCAM) has been reported to be important for the maturation of perisomatic inhibitory synapses in the cortex (Pillai-Nair et al., 2005; Brennaman and Maness, 2008; Chattopadhyaya et al., 2013). NCAM acts through activation of Fyn kinases and possibly recruits other adhesion molecules (Chattopadhyaya et al., 2013). Interestingly, it was recently reported that also members of the *ephrin* family, ephrinA5 and EphA3, can affect inhibitory synapses and they require NCAM for their action (Brennaman et al., 2013). *In vivo*, NCAM is polysialylated (NCAM-PSA) in an experience-dependent manner and developmental downregulation of NCAM-PSA was shown to coordinate maturation of perisomatic inhibitory synapses in the visual cortex (Di Cristo et al., 2007).

Several components of the dystrophin-associated glycoprotein complex (DGC), such as dystroglycan, dystrophin, and dystrobrevin, are also specifically located at a subset of inhibitory synapses (Knuesel et al., 1999; Brünig et al., 2002; Lévi et al., 2002; Grady et al., 2006), but the function of this complex at inhibitory synapses is not well understood. The DGC could be linked to postsynaptic neuroligin-2 via the scaffolding protein S-SCAM (Sumita et al., 2007) and to presynaptic neurexins (Sugita, 2001). Interestingly, a synaptic guanine exchange factor SynArfGEF has been identified that specifically co-localizes at inhibitory synapses, which could be involved in the downstream signaling pathway of the DGC (Fukaya et al., 2011), but its exact function remains to determined.

Integrins are receptors for extracellular matrix proteins, soluble factors, and counter-receptors on adjacent cells and they have an intracellular link to actin filaments via adaptor proteins (Hynes, 2002; Harburger and Calderwood, 2009). Integrins have been implicated in activity-dependent synaptic changes (Chavis and Westbrook, 2001; Chan et al., 2003) and in homeostatic scaling of excitatory synapses (Cingolani et al., 2008). At glycinergic inhibitory synapses in the spinal cord, postsynaptic β1 and β3 integrins have been reported to regulate glycine receptor stabilization at the postsynaptic membrane, with the two integrins acting in opposing directions (Charrier et al., 2010).

Finally, the cell adhesion molecule neurofascin has been shown to regulate the formation of a specific subset of inhibitory synapses on the axon initial segment of principal neurons (Ango et al., 2004; Burkarth et al., 2007; Kriebel et al., 2011).

## ROLE OF SECRETED FACTORS AND RETROGRADE MESSENGERS AT INHIBITORY SYNAPSES

Above we have described how cell adhesion molecules may provide signals to inhibitory axons from direct cell–cell contacts. However, inhibitory synapses may also be affected by signals from more distal origin. Nearby dendrites or surrounding cells can secrete trophic (or anti-trophic) factors, which may affect inhibitory synapse function and/or formation. Indeed, retrograde signals from the postsynaptic dendrite, such as endocannabinoids, nitric oxide (NO) or brain-derived neurotrophic factor (BDNF), or glutamate spillover from nearby excitatory synapses are known to regulate synaptic release at inhibitory synapses during many forms of short-term and long-term plasticity (Heifets and Castillo, 2009; Regehr et al., 2009; Castillo et al., 2011). Here we discuss secreted factors that have been linked to the formation of inhibitory synapses and that might play a role in activity-dependent regulation of the number of presynaptic terminals made by inhibitory axons.

### BRAIN-DERIVED NEUROTROPHIC FACTOR

Brain-derived neurotrophic factor (BDNF) is a secreted neurotrophin that has been shown in many different preparations to promote the formation and maturation of inhibitory synapses by presynaptic modifications (Vicario-Abejón et al., 1998; Huang et al., 1999; Marty et al., 2000; Yamada et al., 2002; Gottmann et al., 2009). Only excitatory neurons produce BDNF (Gottmann et al., 2009; Park and Poo, 2013) and BDNF is released from principal neurons in an activity-dependent manner (Kolarow et al., 2007; Kuczewski et al., 2008; Matsuda et al., 2009), which makes BDNF an attractive candidate molecule to regulate activity-dependent inhibitory synapse formation (Liu et al., 2007). Interestingly, the availability of postsynaptic BDNF signaling in individual neurons was shown to affect the number and strength of inhibitory synapses specifically onto the affected neurons (Ohba et al., 2005; Kohara et al., 2007; Peng et al., 2010). These cell-autonomous effects indicate the potential for BDNF in mediating changes in inhibitory synapses with high synaptic specificity. In excitatory axons, BDNF was shown to reduce the anchoring of synaptic vesicles at presynaptic terminals and thereby increase the exchange of vesicles between boutons (Bamji et al., 2006). It is currently not known if BDNF has a similar effect in inhibitory axons.

### NEUREGULIN1

Neuregulin1 is a neurotrophic factor, which exists in various membrane-bound and diffusible isoforms. Mutations (both loss-of-functions and gain-of-function) in neuregulin1 have been linked to schizophrenia (Mei and Xiong, 2008). The main receptor for neuregulin1, ErbB4, is specifically expressed in interneurons (Vullhorst et al., 2009; Fazzari et al., 2010) and is located at postsynaptic densities of excitatory synapses in interneuron dendrites as well as at inhibitory axon terminals. An important role for neuregulin1 is the regulation of excitatory input onto interneurons through postsynaptic ErbB4 (Fazzari et al., 2010; Wen et al., 2010; Ting et al., 2011). Presynaptic ErbB4 can enhance GABA release from inhibitory synapses (Woo et al., 2007; Fazzari et al., 2010) and may affect the number of synapses made by inhibitory axons (delPino et al., 2013). In addition to ErbB4, neuregulin1 isoforms can also activate other receptors resulting in downregulation of postsynaptic GABA_A_ receptors (Yin et al., 2013). This suggests that neuregulin1 has multiple actions on inhibitory synapses depending on the isoform and activated receptors.

### FGF7

Fibroblast growth factors (FGFs) are secreted signaling glycoproteins, which exert their effect by binding to FGF receptor tyrosine kinases (FGFR). In the brain, FGF signaling is important for several developmental processes, including patterning of different brain structures and neurogenesis (Dono, 2003; Reuss and von Bohlen und Halbach, 2003). In addition, FGFs have been implicated as target-derived presynaptic organizers (Umemori et al., 2004). FGF7 is of particular interest, as it localizes specifically to inhibitory synapses in the hippocampal CA3 region, where it is secreted from the postsynaptic membrane and organizes presynaptic release properties (Terauchi et al., 2010). FGF receptors have been shown to directly interact with adenosine A2A receptors (Flajolet et al., 2008), which are important for GABA release (Cunha and Ribeiro, 2000) as well as for GABA uptake from the synaptic cleft (Cristóvão-Ferreira et al., 2009). In this way, FGFR and A2A receptors may act together to regulate GABAergic transmission in the hippocampus.

### FACTORS FROM GLIA CELLS

Studies with neuronal and astrocyte co-cultures and astrocyte-conditioned medium have shown that astrocyte-released factors are crucial for synapse formation and plasticity (Elmariah et al., 2005; Christopherson et al., 2005a; Hughes et al., 2010; Crawford et al., 2012). For instance, thrombospondins, oligomeric proteins of the extracellular matrix produced by astrocytes (Christopherson et al., 2005b; Eroglu et al., 2009) are involved in the formation of glutamatergic synapses and the pro-inflammatory cytokine TNFα, coming from glia, (Stellwagen and Malenka, 2006) plays a role in homeostatic plasticity of these synapses. In addition, a different and so far unidentified, protein is secreted by astrocytes, which has been found to increase the density of inhibitory synapses in cultured neurons (Elmariah et al., 2005; Hughes et al., 2010).

### GABA

A special secreted factor is the inhibitory neurotransmitter GABA itself. It is well-established that synapse formation does not depend on neurotransmitter release (Verhage, 2000; Harms and Craig, 2005; Schubert et al., 2013). However, the development and maturation of inhibitory synapses are influenced by their neurotransmitter GABA (Li et al., 2005; Huang and Scheiffele, 2008; Huang, 2009; Lau and Murthy, 2012). It was shown that individual axons of parvalbumin-positive basket cells are sensitive to their own GABA release (Chattopadhyaya et al., 2007; Wu et al., 2012) and that the amount of GABA release per vesicle can be regulated by activity (Hartman et al., 2006; Lau and Murthy, 2012). Inhibitory boutons are less dynamic in axons in which GABA release is impaired (Wu et al., 2012) or when GABA receptors are blocked (Schuemann et al., 2013), strongly suggesting that GABA is used as an important activity sensor for regulating activity-dependent presynaptic changes at inhibitory synapses. Both GABA_A_ and GABA_B_ receptors have been implicated in mediating this regulation (Fu et al., 2012; Schuemann et al., 2013), but the precise molecular mechanisms remain unknown.

## OTHER FACTORS

In addition to cell adhesion molecules and secreted factors, there are many other factors that may affect activity-dependent plasticity of inhibitory axons. For instance, it is well-established that extracellular matrix molecules can play a role in the development and maturation of synapses in the central nervous system and specific interactions between cell adhesion molecules and the extracellular matrix have been revealed (Di Cristo et al., 2007; de Wit et al., 2013; Siddiqui et al., 2013). There are a few studies in which the absence or overexpression of extracellular matrix proteins affected inhibitory synapses specifically (Saghatelyan et al., 2001; Nikonenko et al., 2003; Brenneke et al., 2004; Pavlov et al., 2006; Su et al., 2010), but the underlying mechanisms remain largely unknown.

And finally, while it is clear that presynaptic components are continuously shared and exchanged between inhibitory boutons along the axons, it is not clear how exactly these proteins are dispersed along the axonal shaft. Presumably sharing occurs through passive diffusion of presynaptic proteins through the axonal shaft, but intracellular transport of synaptic cargo could also play a role. Axons contain extensive microtubule and actin networks and there are various motor proteins that deliver and ship transport vesicles, potentially affecting the amount of proteins available for exchange and synapse formation at boutons. For instance, it was shown that intra-axonal movement of mitochondria is enhanced when activity is blocked (Goldstein et al., 2008; Cai and Sheng, 2009; Obashi and Okabe, 2013), but it is not clear if this is due to enhanced motor protein activity or decreased anchoring at synapses. Further research on the possible activity-dependent regulation of intracellular transport of synaptic cargo (Guillaud et al., 2008; Maas et al., 2009; MacAskill et al., 2009) will be needed to address this issue in the future.

## CONCLUSION

**FIGURE 3:**
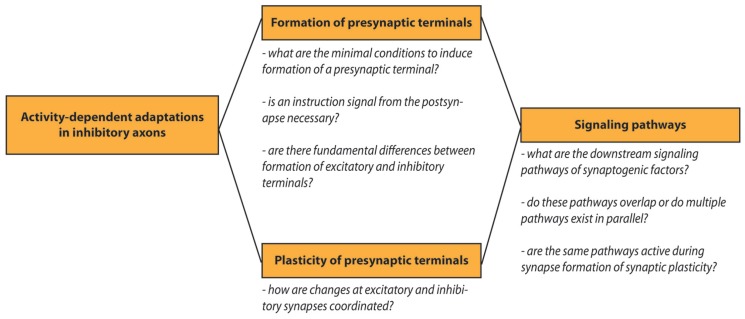
**Outstanding research questions.** Schematic overview of open research questions on activity-dependent adaptations in inhibitory axons.

Research on activity-dependent adaptations in inhibitory axons continues to generate novel insight in the cellular processes of synapse formation and plasticity. Many open questions remain to be answered in the future and we listed a few of these in a small scheme (**Figure 3**). In this review we have painted a picture of the inhibitory axon as a dynamic structure that can quickly adjust to a changing environment, by responding to local signals from postsynaptic cells via adhesion molecules and to global signals from the local neuronal network. A highly dynamic inhibitory system might serve to quickly respond to changes to allow circuit rearrangements by excitatory connections. For a healthy brain changes at inhibitory and excitatory synapses need to be well-coordinated at all times as subtle defects in this coordination can cause defects in circuitry and may underlie psychiatric disorders. This means that the interplay between plasticity of excitatory and inhibitory synapses is an important factor for the stability of neuronal circuits. The precise response of the inhibitory axon will be determined by the combination of internal and external factors, such as the availability of synaptic proteins within the axon, or the combination of the extracellular factors and cell adhesion molecules that are present at the membrane. It will be an important challenge for future research to unravel the precise molecular and cellular mechanisms and to further uncover pathways that affect synapse formation and plasticity of inhibitory synapses.

## Conflict of Interest Statement

The authors declare that the research was conducted in the absence of any commercial or financial relationships that could be construed as a potential conflict of interest.
